# The Artificial Production of Pneumothorax in Pathisis by Injection of Nitrogen
1Read at a Meeting of the Bristol Medico-Chirugical Society held on May 8th, 1912.


**Published:** 1912-06

**Authors:** Hubert Chitty


### THE ARTIFICIAL PRODUCTION OF PNEUMOTHORAX IN PHTHISIS BY INJECTION OF NITROGEN.[1]

BY

HUBERT CHITTY, M.B., M.S., F.R.C.S.,

I FEEL somewhat diffident in writing on a subject of which my own experience is very slight, but thanks to the courtesy of Dr. Campbell-Faill, I am at the present moment treating a

[1] Read at a Meeting of the Bristol Medico-Chirurgical Society held on May 8th, 1912.

couple of patients in conjunction with him, but we commenced our treatment so recently that it would be unfair to draw any conclusions from them as to what the end results may be. The subject has, however, been much before the medical profession of late, and I hope it may serve a useful purpose to summarise the results which others have obtained by this method of treatment.

During the past hundred years it seems to have occurred to a good number of observers that the course of pulmonary tuberculosis might be favourably influenced by mechanical means. Spasmodic attempts have been made to limit the movements of the lungs by strapping the chest, or by compressing it by weights. Of recent years, too, operations have been performed upon the bony chest wall in order to favour the collapse of tuberculous cavities. Other operations have been undertaken with the idea of draining these cavities, or of removing the whole or part of a tuberculous lung. The results of these last procedures have generally proved fatal.

Forlanini noticed that quite a number of cases are on record in which the arrest of pulmonary tuberculosis has coincided with the development of a pleural effusion, and this observation led him (in 1882) to suggest that the induction of an artificial pneumothorax might exercise a beneficial effect on the course of this disease. He himself has reported many cases successfully treated in this way, and the method is being extensively carried out on the continent and in America. In all upwards of four hundred cases have been recorded.

Several observers have practised this operation in Great Britain, but it is only since Dr. Lillingston ably summarised the treatment and its results, in an article in the *Lancet* (1911, ii., 145) last year, that any general interest has been taken in it by the medical profession of this country.

Let us consider the rationale of the proceeding. We know how important a part rest plays in the cure of tuberculosis in other parts of the body. Now it is obvious that if a satisfactory pneumothorax can be produced, the lung on that side will be absolutely immobilised. In addition to this, however, any tuberculous cavities will be encouraged to collapse, and their contents will be expressed. Every doctor knows how essential is free drainage to the cure of an abscess in other parts of the body, and it is only reasonable to suppose that the same principle holds good in the case of the lung. A tuberculous cavity cannot be expected to close all the while it is held open by its attachment to the rigid chest wall, and is distended by purulent material which cannot drain away.

Now, what one sees when a pneumothorax is induced is as follows: During the first few hours the amount of expectoration is greatly increased as the lung empties itself of its retained secretions, and as a rule the temperature rises at this period. Afterwards the temperature falls to normal, the cough diminishes, and the patient soon experiences a distinct feeling of improvement.

One knows how exercise, excitement, or an exacerbation of the cough will send up a tuberculous patient's temperature, or produce marked fluctuations in the opsonic index. Presumably this is by causing the absorption of large doses of bacterial products from the diseased lung. By the induction of a pneumothorax we put the lung at rest and diminish the amount of blood circulating through it, and, as a natural consequence, we find a steadying of both temperature and opsonic index. This must be of great importance if we wish to treat the patient successfully by tuberculin injections. The pulse follows the temperature. In one of our cases it fell from 120 on the morning of the first injection of gas to 80 two days later.

Before describing the operation I will enumerate the principal dangers. You will thus be able to appreciate how all, or almost all, of these may be obviated by careful attention to details of technique. Briefly, then, the chief dangers are:—

(1) Pleural reflex, *i.e.* shock, or even sudden death due to tampering with the pleura. Though this disaster is fortunately extremely rare, yet it may occur, just as it may when one taps a chest for pleurisy, or washes out an empyema cavity.

(2) Gas embolism, from the injection of gas into a vein, instead of into the pleural cavity.

(3) Asphyxia.

(4) Infection, with the production of a pyopneumothorax.

Of the minor complications, faintness during the gas injection, or shortly afterwards, is not uncommon. Dyspnœa for a few hours is the rule. It causes little discomfort. Slight temporary dysphagia has been recorded. Pleural effusion occurs during the course of treatment in 30 per cent. of all cases. One need rarely interfere, but may aspirate and replace by gas if it is causing any unpleasant subjective sensations. Surgical emphysema when it occurs subsides without treatment. It may be prevented by strapping a pad over the site of puncture.

The apparatus, of which I have here a simple home-made example, consists essentially of :—

(1) A bottle containing nitrogen.

(2) A hand bellows connected with

(3) A second bottle filled with water. This water can be forced by the bellows into the first bottle, displacing an equal volume of nitrogen. Both bottles are graduated.

(4) A delivery tube which leads from the nitrogen bottle and is connected by a Y piece with a water or mercury manometer. The gas may be passed through a warming coil if desired.

(5) A needle, of which there are several forms. The commonest form in use is that designed by Saugmann.

*Operation.*

A preliminary injection of morphia or hyoscine lessens the tendency to pleural reflex and shock. I need scarcely mention that the patient's skin, the needle and the operator's hands must be carefully sterilised, as faulty asepsis may lead to a fatal result. The needle is pushed into an intercostal space till it is felt to penetrate the pleura. The point selected will be as far as possible from the chief seat of the disease, and more than one puncture may have to be made before a spot is found free from adhesions. In quite a large percentage of cases no such spot has been discovered, and consequently failure to produce a pneumothorax at all has resulted. It is well to anæsthetise the skin before the initial puncture is made, as one generally uses a large needle at the first time of operating. Brauer dissects down to the pleura by open operation, so as to make certain that the point of the needle is actually introduced between its two layers. He is thus enabled to use considerable force to break down adhesions, and he records remarkably few failures. For my own part I should certainly try simple puncture first. The needle is connected with a manometer, and when one thinks the needle point is in the pleural cavity the manometer is read. If good oscillations are shown (Lillingston mentions —14 cm. of water on inspiration, to —6 cm. during expiration as an average reading) there can be no doubt that the needle has in fact entered the pleural cavity. Small negative oscillations round about —4 cm. of water indicate that the lung has been punctured. If the reading is not satisfactory one of three things has happened: (1) the needle is not between the two pleural layers, (2) these layers are adherent, or (3) the needle is blocked. Under these circumstances it is inadvisable to proceed, for the needle may have punctured a vein, and then there will be the danger of causing gas embolism. To minimise this risk I have used an ordinary trocar and canula for the first puncture. Then, removing the trocar, I introduce an inner canula which has a solid end and a lateral opening, and which projects a short distance beyond the end of the outer canula. It is practically impossible for this to get blocked, or for it to enter a vein. Once a pneumothorax has been produced a fine aspirating needle is all that is required to maintain the condition. Being certain that the needle is in position, proceed slowly to pump in gas. Nitrogen is generally used, as it is not absorbed so quickly as is air. As the gas is introduced a careful watch must be kept on the patient, and if he manifests any unfavourable symptoms a stop should at once be made. At the end of the first injection the pressure is as a rule still negative, but where there are many adhesions the injection of even a few hundred c.c. of gas may cause the pressure to become positive. The initial pressure should not be raised above 5 cm. of water, and except in the case of a partial pneumothorax, in which it is desired to break down adhesions, at no time should it be raised above 10 cm. of water. Probably not more than 200 to 500 c.c. should be injected at the first sitting, though on subsequent occasions the amount may be raised to a litre or more, if this does not seem to be causing distress. The effects of large injections and high pressures may be alarming. Pain may be caused by the tearing down of adhesions; dyspnœa from the mediastinum being pushed over, thus hampering the action of the opposite lung; asphyxia caused by the secretions of the diseased lung being emptied into the trachea, and aspirated into the sound lung. After the initial injection the process may be repeated at intervals of a day or so, till physical signs and X-ray examinations demonstrate complete collapse of the lung. In order to accomplish this a positive pressure of 5 to 10 cm. of water should be produced. Once this has been obtained the condition may be maintained by an injection about once a month.

Let us now consider what are suitable and what unsuitable cases. In cases where there is active and advanced disease on both sides it would obviously be useless and indeed harmful to throw all the work upon one lung. Attempts have been made, it is true, to compress the two lungs alternately, but, at any rate for the present, we may regard advanced bilateral disease as the chief contradiction. Extensive disease in other organs, especially intestinal tuberculosis, would also be a bar to operation. Of the remaining cases we may consider as specially suitable :—

(1) Those in whom the disease is advanced on one side, whilst the opposite lung is *un*affected, *slightly* affected, or quiescent.

(2) Those in whom the temperature remains high in spite of the usual methods of treatment, and who show signs of auto-inoculation whenever they take any exercise. I may here instance one of our own cases, whose evening temperature for at least a year had been from 99 to 100 degrees, in spite of his having spent a long time at a sanatorium, and of the fact that at home he had been living in a shelter. His temperature came down to normal within forty-eight hours, and has remained at that level ever since. Numerous similar instances have been recorded.

(3) Cases which are going downhill in spite of the usual methods of treatment.

(4) Early unilateral cases for whom sanatorium treatment is not available. Especially does this apply to the bread-winner of the family.

(5) Cases of severe recurrent hæmoptysis.

One is often in doubt as to which side is giving rise to the bleeding, but in these cases it would be quite justifiable to compress the worse lung, and, if this had no effect, then to aspirate the gas and repeat the operation on the other side.

(6) Although most of the recorded cases have been patients suffering from chronic tuberculosis, yet this has not been by any means invariably so, and some cases of acute phthisis have been successfully dealt with.

Laryngeal tuberculosis does not appear to be a bar to the operation. In fact, several recoveries from this form of disease have been recorded. This is easy to understand, as the cessation of cough, and of the constant passage of tuberculous sputum over the larynx must be beneficial.

The question naturally arises, "What is the duration of the treatment?" No very definite answer can yet be given. There is little doubt that in advanced cases it must be prolonged for eighteen months to two years; but in very early cases cure has been reported when the gas has been allowed to absorb completely after a few months only. If the treatment has not been sufficiently prolonged recrudescence has been the rule. Under these circumstances it has been found impossible to reproduce the pneumothorax owing to the formation of dense adhesions between the two pleural layers. Therefore when it has been decided to allow the lung to re-expand a careful watch should be kept on the patient. A rise of temperature or any increase in the amount of expectoration would suggest the advisability of keeping the lung at rest for a few months longer.

It is wonderful how readily the healthy portions of the lung will expand and fill the thoracic cavity, even after the treatment has lasted from two to three years.

In discussing the results of treatment we must bear in mind that all, or almost all, the reported cases have been of one type, viz. patients suffering from very advanced disease, in whom other methods had proved incapable of arresting the morbid process, and in whom the mortality would most likely have been at least 90 per cent. Many cases have been too recently published for us to be able to judge what their ultimate fate may be, but undoubtedly the initial results have been full of promise. Certainly well over one hundred cases have, however, been recorded in whom the treatment was commenced many years ago, and in these there would seem to have been a permanent arrest of the disease in at least 60 per cent. In many of the more recent cases immense improvement has already ensued, and patients who a few months back seemed almost moribund now appear to be on the high road to recovery. Where deaths have been recorded during the course of the treatment they have been due, as a rule, to disease in the other lung, to tuberculous disease in some other part of the body, or to some intercurrent malady. In the few cases, in which *post-mortem* examinations have been made, healing by fibrosis has generally been recorded, though this has not always been evident.

One theoretical objection has been raised to the treatment, viz. that when a *spontaneous* pneumothorax occurs in a late case of phthisis a fatal result is frequent, but I hope that I have shown this to be quite a specious argument in that the two conditions are in no way comparable. In a spontaneous case there is no regulation of the pressure, and no stopping when symptoms of shock arise. Moreover, the pleural cavity is in direct communication with the lung, and is very liable to become infected from this diseased organ.

I hope I have shown that this method of treatment has a future before it, even though it be for only a limited number from out the vast array of the tuberculous. There is plenty of room for experiment and observation, and I trust that many will give this treatment a trial upon certain selected cases. Risk there may be, but who counts risk when life and death are in the balance?

Dr. D. KENNEDY (introduced) said: "I do not think Dr. Chitty sufficiently emphasised the dangers and difficulties met with in attempting to produce an artificial pneumothorax. The experience of surgeons who have done a large number of cases is that in about one-third of them it is impossible to perform the operation, owing to adhesions between the two layers of the pleura. This complication occurred in the first case we attempted at Nordrach, in which twelve punctures in all were made on three separate days. One must also be able to recognise when the point of the needle is in a vein, or if it has gone through the pleura into the lung; in the latter case the oscillations of the manometer (water) are usually about from $-2$ and $+2$ cm. If in doubt as to whether the needle is in the lung or not, a few drops of peppermint or eau-de-Cologne added to the jar containing the nitrogen will soon settle matters, as on injecting a small quantity of gas the peppermint or eau-de-Cologne, as the case may be, will be evident from the patient's breath. No harm appears to have resulted from injecting gas into the lung.

"Our first case was, as I mentioned before, unsuccessful, and the second was a very difficult one, the patient having a large cavity in the upper lobe, and although only a very limited pneumothorax could be produced, still the expectoration diminished to about one-fifth almost at once, the cough became much less, and the temperature fell several points. The pressure after each injection in this case was very high, $+17\frac{1}{2}$ and 19 cm. The patient only experienced very slight dyspnœa, but had a difficulty in swallowing solid food for a few days after each injection. Diffuse surgical emphysema occurred after the second injection, but this was subsequently prevented by strapping a piece of gauze tightly over the puncture.

"The third case, which we did a few days ago, has been perfectly straightforward from the first. This patient also had a large cavity in the upper lobe, and had had a couple of very severe hemorrhages just previously. The manometer showed a pressure of $-3\frac{1}{2}$ and $-5$ cm. After injecting 300 cc. of nitrogen, pressure was $-\frac{1}{2}$ and $-3$ cm. The temperature in this case did not fall until the lung was practically completely collapsed; this is, I believe, in accordance with the experience of other observers.

"The needles we use are Saugmann's, which have the opening at the point, and Lillingston's modification which have the opening at the side near the point—they are about the size of a knitting needle. A stilette has frequently to be used, as the lumen gets blocked in passing through the tissues."

(See also p. 189, a communication from Dr. Mariette).

